# Clinical and molecular features of candidemia: a three-year retrospective study in Northern Guizhou, China

**DOI:** 10.3389/fcimb.2025.1700990

**Published:** 2026-01-05

**Authors:** Xianlian Chen, Pujing Nie, Xiandan Chen, Changjin Liu, Guangli Wang, Yanfeng Peng, Shilu Luo, Tao Chen, Huan Zhou, Xun Min, Jian Huang

**Affiliations:** 1 Department of Laboratory Medicine, Affiliated Hospital of Zunyi Medical University, Zunyi, Guizhou, China; 2 School of Laboratory Medicine, Zunyi Medical University, Zunyi, Guizhou, China; 3 Department of Dermatology, Affiliated Hospital of Zunyi Medical University, Zunyi, Guizhou, China; 4 Department of Laboratory Medicine, Renji Hospital, School of Medicine, Chongqing University, Chongqing, China

**Keywords:** candidemia, clinical characteristics, prognostic risk factors, antifungal susceptibility, multilocus sequence typing

## Abstract

**Objective:**

Candidemia, a life-threatening infection with rising incidence and substantial mortality, necessitates improved management strategies. This study aimed to investigate the clinical features, species distribution, risk factors, antifungal susceptibility, and molecular epidemiology of *Candida albicans* isolates from candidemia patients over a three-year period.

**Methods:**

This retrospective study included 133 patients with candidemia from a teaching hospital in Guizhou, China between December 2019 and November 2022. Clinical data were compared between *C. albicans* (n = 63) and non-*C. albicans* (NAC) (n = 70) groups. Risk factors and prognostic biomarkers were identified using logistic regression and ROC analysis. Multilocus sequence typing (MLST) was performed for *C. albicans* isolates.

**Results:**

NAC species predominated (52.6%) over *C. albicans* (47.4%). The 30-day all-cause mortality was 48.1%, higher in *C. albicans* infections. For *C. albicans*, hypoalbuminemia and septic shock were independent mortality risk factors, while antifungal therapy and higher platelets were protective. For NAC, septic shock and elevated serum urea were risk factors. Prognostic biomarkers included D-dimer and CRP for *C. albicans*, and serum urea and total bilirubin for NAC. Most isolates were antifungal-susceptible, though *C. glabrata* and *C. tropicalis* showed increased azole non-susceptibility. MLST of 48 C*. albicans* isolates identified 7 novel alleles and 23 new DST types. CC9 was the predominant clonal complex. CC138 and CC139 are newly reported clonal complexes.

**Conclusion:**

This study confirms that candidemia remains a serious threat with high mortality. The distinct risk factors and prognostic biomarkers between *C. albicans* and non-*C. albicans* species indicate the need for species-specific management. Although overall resistance remains low, emerging azole non-susceptibility in key species underscores the necessity for continuous susceptibility surveillance. MLST revealed a highly diverse and evolving *C. albicans* population, including novel genetic lineages. Integrating molecular epidemiology with clinical data is crucial for advancing global candidemia management.

## 1 Introduction

Candidemia represents the most common clinical manifestation of invasive candidiasis (IC) (Pfaller and Diekema, 2007). Over the past two decades, its incidence has increased by 3.5-fold (Asmundsdóttir et al., 2005). In the United States, *Candida* ranks as the fourth most common cause of hospital-acquired bloodstream infections, accounting for approximately 9% of all pathogenic isolates (Wisplinghoff et al., 2004). Recent studies further indicate that *Candida* has become the predominant pathogen in hospital-acquired bacteremia in the U.S., with its prevalence surpassing that of several common bacterial species, including *Staphylococcus aureus*, *Escherichia coli*, *Klebsiella pneumoniae*, and *Pseudomonas aeruginosa* (Magill et al., 2014). Candidemia is associated with high mortality rates, ranging from 35% to 75% (Gudlaugsson et al., 2003; Pfaller and Diekema, 2007; Yapar, 2014; Duggan et al., 2015), posing a substantial threat to patient prognosis. Owing to its significant morbidity and mortality burden, prolonged hospital stays, and increased healthcare costs, candidemia has emerged as a critical challenge in the field of infection control (Morgan et al., 2005; Smith et al., 2007; Oren and Paul, 2014). The convergence of several healthcare trends has led to a growing population at risk for candidemia. This includes more patients with iatrogenic immunosuppression (e.g., post-transplantation), the widespread use of invasive devices that create portals of entry, and broad-spectrum antibiotic use that promotes fungal colonization (Kullberg and Arendrup, 2015). Consequently, common risk factors now prominently include intravenous catheterization, prolonged ICU stays, organ transplantation, parenteral nutrition, and exposure to broad-spectrum antibiotics in immunocompromised patients (Yapar, 2014; Çetin et al., 2024).

*Candida albicans* remains the most frequently isolated species in candidemia. However, the proportion of non-*Candida albicans* (NAC) infections has been gradually increasing in recent years. This epidemiological shift may result from widespread azole use, an increasing number of immunocompromised hosts, prolonged ICU stays, frequent invasive procedures, and expanded immunosuppressive therapy. NAC species often exhibit intrinsic or acquired resistance to commonly used antifungal agents, thereby presenting greater therapeutic challenges. Although *C. albicans* demonstrates a relatively lower rate of drug resistance (Liu et al., 2014), it is associated with mortality comparable to that of NAC species (Dutta and Palazzi, 2011) and has occasionally been linked to nosocomial outbreaks (Asmundsdóttir et al., 2008; Hammarskjöld et al., 2013). Therefore, a comprehensive understanding of the species distribution, prognostic risk factors, antifungal susceptibility profiles, and molecular epidemiological characteristics of candidemia is essential for optimizing therapeutic strategies and guiding infection control measures. Multilocus sequence typing (MLST) is a high-resolution genotyping technique based on single nucleotide polymorphisms (SNPs) in internal fragments of housekeeping genes. It has been widely used for analyzing genetic diversity and conducting epidemiological studies in *Candida* species (Shin et al., 2011). Strain data from different laboratories and geographical origins can be compared via online MLST databases. This method provides valuable insights into strain diversity, mechanisms of drug resistance acquisition, nosocomial transmission dynamics, and infection control strategies (Tsai et al., 2015; Byun et al., 2018; Boonsilp et al., 2021).

In this study, we aimed to describe the clinical characteristics, species distribution, prognostic risk factors, and antifungal drug sensitivity of patients with candidemia, and to conduct MLST analysis on the genetic correlation of *C. albicans* isolated from candidemia in a teaching hospital in Guizhou over a period of three years.

## 2 Materials and methods

### 2.1 Study setting, case definitions, and data collection

The study was conducted at a teaching hospital in northern Guizhou, China, with 2800 beds across 7 ICU and 56 general wards. Patients aged ≥18 years with *Candida* isolated from blood cultures between December 2019 and November 2022 were enrolled. Clinical data, including demographics, comorbidities, risk factors, and treatment regimens, were extracted from hospital records, and the 30-day all-cause mortalities post-diagnosis were recorded. Candidemia was defined per the 2019 EORTC/MSG consensus criteria (Donnelly et al., 2020). Episodes were considered distinct if separated by ≥1 month or involving different *Candida* species (Sandven et al., 1998). Clonal complex (CC) was defined as a group of isolates with high genetic similarity that share a recent common ancestor (Aanensen and Spratt, 2005). This work was approved by the Ethics Committee of the Affiliated Hospital of Zunyi Medical University (Approval No.: KLLY-2022-028).

### 2.2 Yeast identification and antifungal susceptibility testing

Isolates were cultured using the BD BACTEC FX200 system and identified via matrix-assisted laser desorption/ionization time-of-flight mass spectrometry (MALDI-TOF MS) (bioMérieux, France) (Buchan and Ledeboer, 2013). Antifungal susceptibility testing was performed using Sensititre YeastOne YO10 panels (Thermo Scientific, USA) for nine drugs. Minimum inhibitory concentrations (MICs) were interpreted per CLSI M27-S4 guidelines and species-specific breakpoints (Song et al., 2020b). Cross-resistance is defined as the phenomenon where different antimicrobial agents within the same class exhibit similar resistance due to a shared mechanism of resistance (George, 1996).

### 2.3 Multilocus sequence typing and phylogenetic analysis of *C. albicans*

Forty-eight *C. albicans* isolates were genotyped using a seven-locus Multilocus Sequence Typing (MLST) scheme (Bougnoux et al., 2003). Allele sequences were submitted to the *C. albicans* MLST database for DST assignment. Unassigned allele sequences were submitted to the MLST database (https://pubmlst.org/organisms/*candida-albicans*), and new allele numbers, including new DSTs, were provided by the curator. Phylogenetic trees were constructed using UPGMA in MEGA7 (Wang et al., 2020), and clonal complexes (CCs) were identified via goeBURST in PHILOVIZ 2.0 (Nascimento et al., 2017). A minimum spanning tree was generated using GrapeTree. To evaluate the proportion of infections caused by clonal isolates, a nosocomial clone was defined as identification of genetically closely related isolates from two or more patients within a period of 90 days (Asmundsdóttir et al., 2008; Tsai et al., 2015).

### 2.4 Statistical analysis

Data were analyzed using SPSS 29.0. Non-normally distributed variables were reported as median (IQR) and compared via Mann–Whitney U tests. Categorical data were analyzed using chi-square or Fisher’s exact tests. Logistic regression identified prognostic factors, and receiver operating characteristic (ROC) curves evaluated diagnostic markers. Significance was set at *P* < 0.05.

## 3 Results

### 3.1 Clinical characteristics and prognostic factors

A total of 133 patients were evaluated to collect data for predicting clinical outcomes. Among the 133 patients included in the analysis (with an average age of 59.9 ± 15.8 years, and 66.2% being male), the incidence of candidemia was 0.32 cases/1, 000 admissions. The 30-day all-cause mortality rate in the study cohort was 48.1%. *C. albicans* (47.4%) was the most prevalent species, followed by *Candida parapsilosis* (18.8%), *C. tropicalis* (17.3%), *C. glabrata* (13.5%), and other minor *Candida* species (3%). The baseline clinical characteristics of 133 patients, stratified by *C. albicans* and NAC candidemia, are summarized in **Table 1**. Significant differences were observed between the two groups regarding age (*P* = 0.021), 30-day all-cause mortality rate (*P* = 0.003), underlying diseases (hypertension (*P* = 0.023), pulmonary infection (*P* = 0.015), cardiovascular disease (*P* = 0.002)), risk factors (urinary catheterization (*P* = 0.018), central venous catheterization (CVC) (*P* = 0.001), hemodialysis (*P* = 0.044), septic shock (*P* = 0.011)), and laboratory parameters, including white blood cell count (WBC) (*P* = 0.006), neutrophil count (*P* = 0.004), and red cell distribution width (RDW) (*P* = 0.043).

**Table 1:** Comparative analysis of clinical features and laboratory indicators in patients with *Candida albicans* versus non-*Candida albicans* candidemia.

	*Candida albicans* (N = 63) (47.4%)	non-*Candida albicans* (N = 70) (52.6%)	*P*-value
Gender (male/female)	42 (66.7%) / 21 (33.3%)	46 (65.7%) / 24 (34.3%)	0.908
Age (years)	66.00 (55.00-72.00)	57.50 (47.50-69.00)	0.021^▲^
ICU hospitalization	43 (68.3%)	43 (61.4%)	0.411
Previous antibiotics exposure	49 (77.8%)	53 (75.7%)	0.779
Blood transfusion	40 (63.5%)	44 (62.9%)	0.940
Parenteral nutrition	40 (63.5%)	35 (50.0%)	0.117
Surgery	36 (57.1%)	40 (57.1%)	1.000
Urinary catheterization	58 (92.1%)	54 (77.1%)	0.018^▲^
Drainage tube	40 (63.5%)	43 (61.4%)	0.806
Bacterial infection	38 (60.3%)	50 (71.4%)	0.176
CVC	63 (100.00)	58 (82.9%)	0.001^▲^
Endotracheal intubation	45 (71.4%)	44 (62.9%)	0.294
Mechanical ventilation	46 (73.0%)	43 (61.4%)	0.156
Hemodialysis	18 (28.6%)	10 (14.3%)	0.044^▲^
Hypoalbuminemia	45 (71.4%)	53 (75.7%)	0.575
MODS	18 (28.6%)	12 (17.1%)	0.115
Autoimmune diseases	2 (3.2%)	3 (4.3%)	1.000
Hypertension	28 (44.4%)	18 (25.7%)	0.023^▲^
Diabetes	23 (36.5%)	20 (28.6%)	0.329
Pulmonary infection	57 (90.5%)	52 (74.3%)	0.015^▲^
Liver disease	8 (12.7%)	10 (14.3%)	0.789
Cardiovascular disease	28 (44.4%)	14 (20.0%)	0.002^▲^
Neurological disease	23 (36.5%)	17 (24.3%)	0.125
Organ tumors	2 (3.2%)	7 (10.0%)	0.118
Urinary tract infection	25 (39.7%)	27 (38.6%)	0.896
Abdominal infection	20 (31.7%)	16 (22.9%)	0.249
Septic shock	40 (63.5%)	29 (41.4%)	0.011^▲^
30-day all-cause mortality	39 (61.9%)	25 (35.7%)	0.003^▲^
WBC (10^9^/L)	11.37 (7.79-14.70)	7.66 (4.74-14.13)	0.006^▲^
Neutrophil count (10^9^ /L)	9.57 (6.20-14.19)	6.73 (3.81-11.61)	0.004^▲^
RDW	15.94 ± 2.85	15.09 ± 1.89	0.043^▲^

WBC, white blood cells; RDW, red blood cell distribution width; CVC, central venous catheterization; MODS, multi-organ dysfunction syndrome; ^▲^*P* < 0.05.

Univariate and multivariate logistic regression analyses were performed to identify risk factors for 30-day all-cause mortality in patients infected with *C. albicans*, with results presented in **Table 2**. Based on the full multivariate model, hypoalbuminemia (odd ratio [OR]: 32.635; 95% confidence interval [CI]: 1.829–582.248; *P* = 0.018), septic shock (OR: 77.050; 95% CI: 2.403–2470.406; *P* = 0.014) and elevated C-reactive protein (CRP) levels (OR: 1.026; 95% CI: 1.001–1.052; *P* = 0.043) were independent predictors of 30-day all-cause mortality. In contrast, antifungal treatment (OR: 0.025; 95% CI: 0.002–0.397; *P* = 0.009) and platelet counts (OR: 0.986; 95% CI: 0.974–0.999; *P* = 0.028) emerged as protective factors. The results of univariate and multivariate logistic regression analyses of risk factors associated with 30-day all-cause mortality in patients with infections caused by NAC species are presented in **Table 3**. Septic shock (OR: 17.373; 95% CI: 2.022–149.282; *P* = 0.009) and increased serum urea levels (OR: 1.081; 95% CI: 1.006–1.161; *P* = 0.034) were independent predictors for 30-day all-cause mortality.

**Table 2:** Univariate and multivariate logistic regression analyses of prognostic factors for 30-day all-cause mortality in patients with candidemia due to *C. albicans*.

Characteristic	Survivors (N = 24)	Non-survivors (N = 39)	Univariate analysis	Multivariate analysis	
			*P*-value	OR (95%CI)	*P*-value
Hypoalbuminemia	12 (50%)	33 (84.6%)	0.003	32.635 (1.829-582.248)	0.018^▲^
Abdominal infection	3 (12.5%)	17 (43.59%)	0.010	4.253 (0.233-77.501)	0.328
Parenteral nutrition	10 (41.67%)	30 (76.92%)	0.005	20.211 (0.870-469.449)	0.061
Urinary catheterization	20 (83.33%)	38 (97.44%)	0.044	240.969 (0.143-406612)	0.148
Endotracheal intubation	13 (54.17%)	32 (82.05%)	0.017	0.478 (0.005-44.023)	0.749
Mechanical ventilation	13 (54.17%)	33 (84.62%)	0.008	0.733 (0.007-78.178)	0.896
Septic shock	7 (29.17%)	33 (84.62%)	<0.001	77.050 (2.403-2470.406)	0.014^▲^
Antifungal treatment^a^	19 (79.17%)	15 (38.46%)	0.002	0.025 (0.002-0.397)	0.009^▲^
Lymphocyte (10^9^/L)	0.87 (0.44-1.06)	0.53 (0.34-0.83)	0.034	9.087 (0.132-624.112)	0.306
D-Dimer	1.71 (0.81-3.55)	3.41 (1.92-6.66)	0.046	1.781 (0.933-3.401)	0.080
Albumin	32.90 (30.2-35.05)	29.10 (25.7-33.3)	0.014	0.921 (0.740-1.145)	0.457
Platelet counts (10^12^/L) ^a^	228.50 (161.75-373.25)	133.00 (58-226)	0.002	0.986 (0.974-0.999)	0.028^▲^
CRP	80.27 (40.22-115.74)	128.00 (65.37-176.93)	0.020	1.026 (1.001-1.052)	0.043^▲^

CRP, c-reactive protein; ^▲^*P* < 0.05; ^a^ is the protective factor.

**Table 3:** Univariate and multivariate logistic regression analyses of prognostic factors for 30-day all-cause mortality in patients with candidemia due to non-*Candida albicans*.

Characteristic	Survivors (N = 45)	Non-survivors (N = 25)	Univariate analysis	Multivariate analysis	
			*P*-value	OR (95%CI)	*P*-value
Gender(male)	34 (75.6%)	12 (48%)	<0.001	14.205(0.786-256.671)	0.072
Hypoalbuminemia	30 (66.7%)	23 (92%)	0.018	0.788(0.036-17.097)	0.880
Parenteral nutrition	17 (37.8%)	18 (72%)	0.006	2.737(0.433-17.293)	0.284
MODS	4 (8.9%)	8 (32%)	0.014	0.535(0.055-5.180)	0.589
Hypertension	7 (15.6%)	11 (44%)	0.009	4.254(0.644-28.122)	0.133
Diabetes	9 (20%)	11 (44%)	0.033	4.876(0.609-39.035)	0.136
Urinary catheterization	30 (66.7%)	24 (96%)	0.005	0.240(0.004-14.224)	0.493
CVC	34 (75.6%)	24 (96%)	0.030	6.204(0.175-219.546)	0.316
Septic shock	8 (17.8%)	21 (84%)	<0.001	17.373(2.022-149.282)	0.009^▲^
WBC (10^9^/L)	6.93 (4.77-11.94)	10.93(4.66-19.23)	0.028	1.882(0.919-3.852)	0.084
Neutrophil count (10^9^/L)	5.48 (3.78-10.43)	9.57(4.06-16.56)	0.042	0.533(0.251-1.134)	0.102
Urea	6.75 (4.26-9.58)	13.92(8.95-21.6)	0.006	1.081(1.006-1.161)	0.034^▲^
Total bilirubin	13.3 (9.8-20.6)	18.3 (12.9-42.35)	0.030	1.021(0.997-1.046)	0.081

WBC, white blood cells; CVC, central venous catheterization; MODS, multi-organ dysfunction syndrome; ^▲^*P* < 0.05.

**Figure 1:**
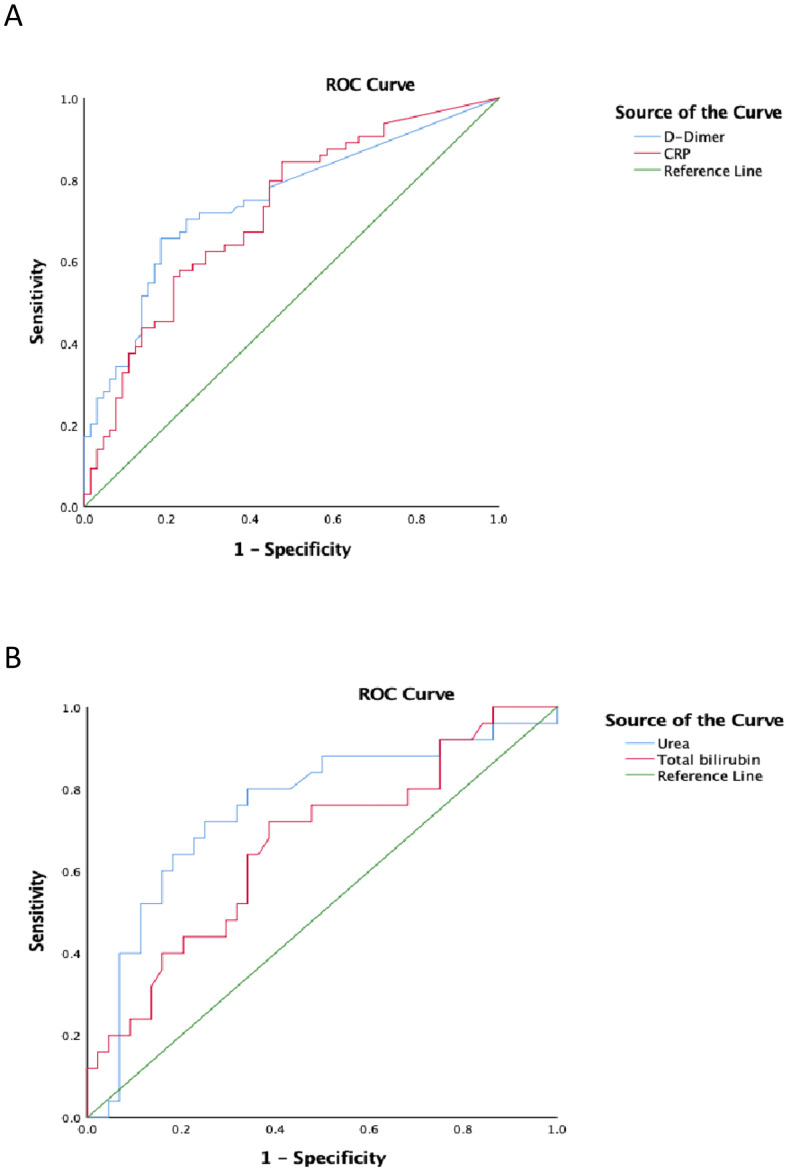
Receiver operating characteristic (ROC) analysis for patients with candidemia, **(A)***Candida albicans*; **(B)** non-*Candida albicans*.

ROC analysis using serum biomarkers that differed significantly between patients who survived and those who did not was performed to assess the ability of serum markers to serve as prognostic indicators for candidemia. Serum D-dimer (area under the curve [AUC] 0.758, 95% CI 0.637–0.879, *P* = 0.001) possessed a higher prognostic value in patients with *C. albicans* and candidemia than other parameters (**Figure 1A**). The critical serum value for survival vs. death was 1.88 µg/mL, with a sensitivity of 66.7%, and a specificity of 87.0%. Serum CRP (AUC 0.756, 95% CI 0.625–0.879, *P* = 0.001) was also valuable in predicting the prognosis of *C. albicans* candidemia (**Figure 1A**). Additionally, serum urea (AUC 0.752, 95% CI 0.632–0.880, *P* = 0.001) and total bilirubin (AUC 0.662, 95% CI 0.528–0.797, *P* = 0.026) were valuable in predicting the prognosis of NAC candidemia (**Figure 1B**).

### 3.2 Antifungal susceptibility

The susceptibility profiles of 108 isolates to nine antifungal drugs are presented in **Table 4**. All isolates remained susceptible to amphotericin B. All isolates besides three *C. glabrata* strains were susceptible to echinocandins (anidulafungin, micafungin, and caspofungin), with the exception of three *C. glabrata* strains. For fluconazole, 74.1% (80/108) of *Candida* isolates were susceptible. Susceptibility-dose dependence (SDD) to fluconazole was observed in all *C. glabrata* isolates and 35.3% (6/17) of *C. tropicalis* isolates. Resistance to fluconazole was detected in *C. albicans* (2.1%; 1/48), *C. parapsilosis* (4.0%; 1/25), and *C. tropicalis* (11.8%; 2/17).

**Table 4:** Susceptibility of the 108 *candida* isolates to nine antifungal agents.

Characteristic	MIC (mg/L)	S/WT (%)	No. of strains (%)					Sensitivity		
			S	SDD	I	R		WT (%)	NWT (%)	
*C. albicans* (n = 48)									
Amphotericin B	≤ 0.12 to 1	100	–	–	–	–	48 (100)		–
Fluconazole	≤ 0.12 to 16	97.9	47 (97.9)	–	–	1 (2.1)	–		–
Voriconazole	≤ 0.008 to 0.5	95.8	46 (95.8)	–	2 (4.2)	–	–		–
Posaconazole	≤ 0.008 to 0.06	100	–	–	–	–	48 (100)		–
Itraconazole	≤ 0.015 to 0.25	100	–	–	–	–	48 (100)		–
Caspofungin	≤ 0.008 to 0.25	100	48 (100)	–	–	–	–		–
Micafungin	≤ 0.008 to 0.3	100	48 (100)	–	–	–	–		–
Anifungin	≤ 0.015 to 0.06	100	48 (100)	–	–	–	–		–
5-Flucytosine	≤ 0.06 to >128	87.5	–	–	–	–	42 (87.5)		6 (12.5)
*C. parapsilosis* (n = 25)									
Amphotericin B	≤ 0.12 to 0.5	100	–	–	–	–	25 (100)		–
Fluconazole	≤ 0.12 to 16	96	24 (96)	–	–	1 (4)	–		–
Voriconazole	≤ 0.008 to 0.5	96	24 (96)	–	1 (4)			–	–
Posaconazole	≤ 0.008 to 0.12	100	–	–	–	–		25 (100)	–
Itraconazole	≤ 0.015 to 0.25	100	–	–	–	–		25 (100)	–	
Caspofungin	0.03 to 1	100	25 (100)	–	–	–		–	–	
Micafungin	0.015 to 1	100	25 (100)	–	–	–		–	–	
Anifungin	0.06 to 2	100	25 (100)	–	–	–		–	–	
5-Flucytosine	≤ 0.06 to 0.25	100	–	–	–	–		25 (100)	–	
*C. glabrata* (n = 18)										
Amphotericin B	0.25 to1	100	–	–	–	–		18 (100)	–	
Fluconazole	1 to16	0	–	18 (100)	–	–		–	–	
Voriconazole	0.03 to 0.5	77.8	–	–	–	–		14 (77.8)	4 (22.2)	
Posaconazole	≤ 0.008 to 2	88.9	–	–	–	–		16 (88.9)	2 (11.1)	
Itraconazole	0.12 to 2	100	–	–	–	–		18 (100)	–	
Caspofungin	0.03 to 0.25	88.9	16 (88.9)	–	2 (11.1)	–		–	–	
Micafungin	≤ 0.008 to 0.5	88.9	16 (88.9)	–	1 (5.55)	1 (5.55)		–	–	
Anifungin	≤ 0.015 to 0.5	94.4	17 (94.4)	–	–	1 (5.6)		–	–	
5-Flucytosine	≤ 0.015 to 4	100	–	–	–	–		18 (100)	–	
*C. tropicalis* (n = 17)										
Amphotericin B	0.5 to 1	100	–	–	–	–		17 (100)	–	
Fluconazole	1 to > 512	52.9	9 (52.9)	6 (35.3)	–	2 (11.8)		–	–	
Voriconazole	0.06 to >16	35.3	6 (35.3)	–	9 (52.9)	2 (11.8)		–	–	
Posaconazole	0.06 to >16	5.8	–	–	–	–		1 (5.8)	16 (94.2)	
Itraconazole	0.12 to > 32	88.2	–	–	–	–		15 (88.2)	2 (11.8)	
Caspofungin	0.03 to 0.25	100	17 (100)	–	–	–		–	–	
Micafungin	0.015 to 0.3	100	17 (100)	–	–	–		–	–	
Anifungin	≤ 0.015 to 0.25	100	17 (100)	–	–	–		–	–	
5-Flucytosine	≤ 0.06 to 0.12	100	–	–	–	–		17 (100)	–	

Notably, cross-resistance was observed in several isolates: 1 C*. glabrata* isolate exhibited cross-resistance among the echinocandins anidulafungin, micafungin, and caspofungin; 14 isolates (8 C*. tropicalis*, 4 C*. glabrata*, 1 C*. albicans*, and 1 C*. parapsilosis*) showed cross-resistance between the fluconazole and voriconazole; and 2 C*. tropicalis* isolates displayed cross-resistance to all tested azoles (fluconazole, voriconazole, posaconazole, and itraconazole). Additionally, 2 C*. glabrata* isolates were resistant to both azole and echinocandin antifungals.

### 3.3 MLST genotyping and genetic diversity of *C. albicans*

**Figure 2:**
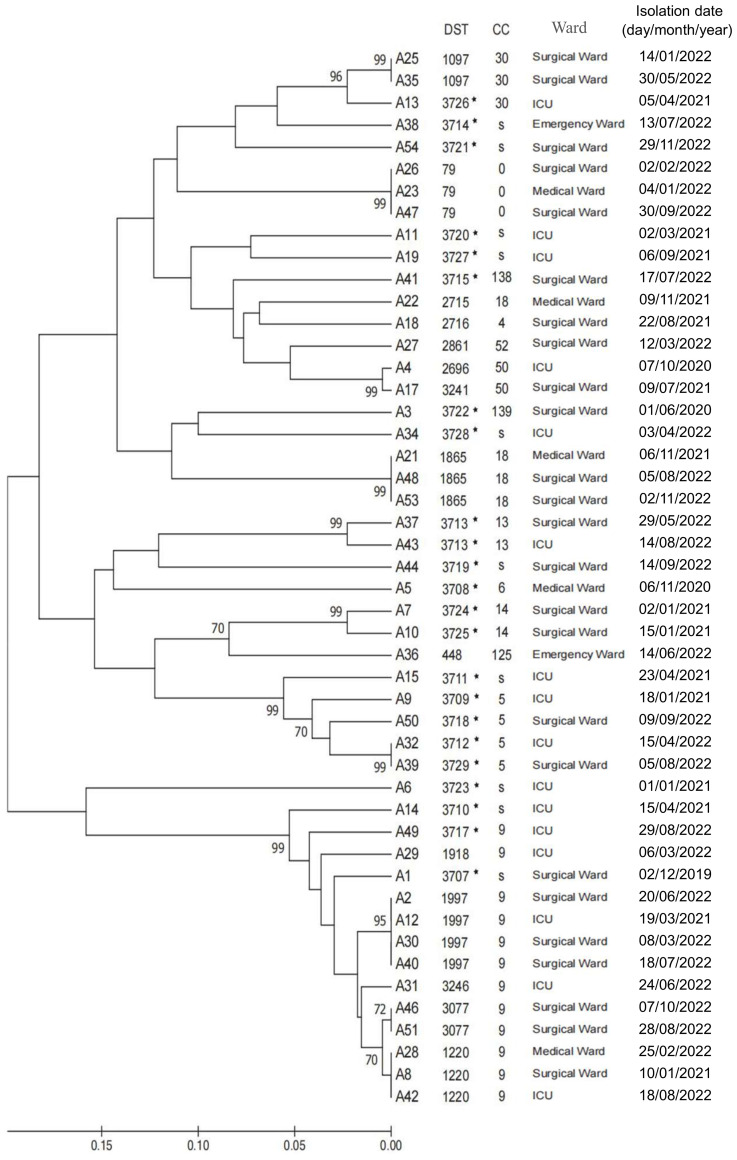
UPGMA dendrogram indicating similarities between 48 C*. albicans* isolates determined using multilocus sequence typing (MLST) of 37 diploid sequence types (DSTs); The CCs determined by goeBURST, S represent singletons, * is new DSTs.

Forty-eight *C. albicans* isolates from patients with candidemia underwent genotyping using the *C. albicans* MLST scheme. DNA sequences from fragments of seven housekeeping genes were concatenated, yielding a 2, 883-bp dataset per isolate. A total of 111 different alleles were detected across the MLST loci investigated, of which 7 were new alleles. The allelic diversity found at these loci resulted in 37 unique MLST genotypes, of which 23 (62.2%) were novel. All were submitted to the *C. albicans* MLST database (DST3707–DST3729) (**Figure 2**). Of these 37 DSTs, 30 (81.1%) were singletons, and 7 were shared by 19 (39.6%) isolates. Analysis of the unrooted dendrogram derived from MLST data revealed that the 48 isolates clustered into 15 major groups and 9 singleton strains. Of these 15 clonal complexes (CCs), 8 were shared by 31 isolates (64.6%). 24 (50%) isolates were categorized into 6 nosocomial clonal complexes (CCs) by our definition. Among these, CC9 was the largest, comprising the most isolates, and demonstrated the longest persistence (**Figure 2**).

**Figure 3:**
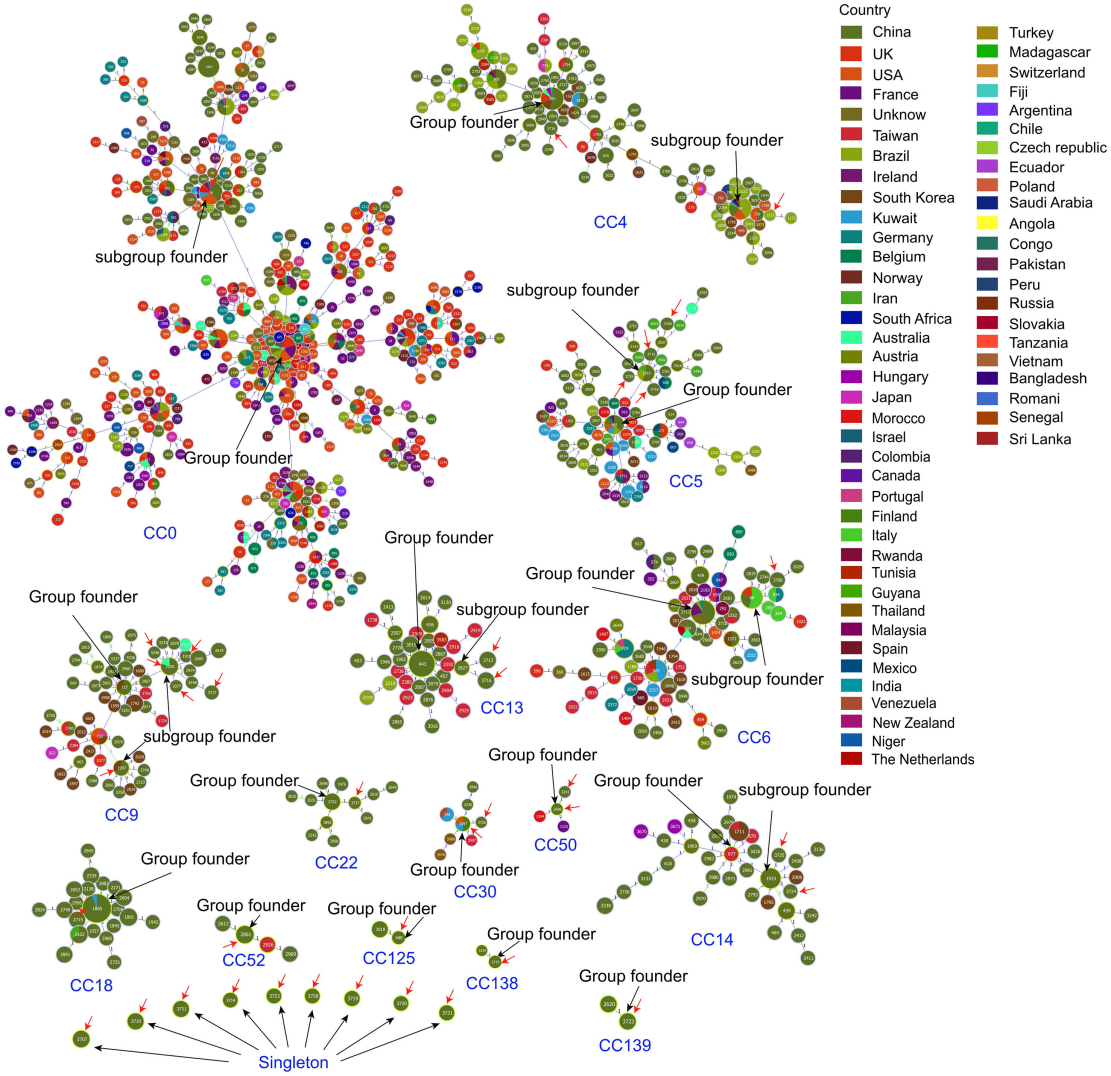
Details of the 15 goeBURST clonal complexes involved in this study; Each circle corresponds to a DST. The size of the circle indicates the number of isolates belonging to the DST. Different colors of the sectors in the circle represent sample sources from different countries. All DSTs in this study are indicated using red arrows, while Group founders and Sub-group founders in each clonal complex (CC) are labeled using black arrows and texts.

We employed the goeBURST algorithm to assess the phylogenetic relationships among the isolates against 3, 882 reference sequence types (DSTs) in the MLST database. The 15 clonal complexes (CCs) (CC0, CC4, CC5, CC6, CC9, CC13, CC14, CC18, CC22, CC30, CC50, CC52, CC125, CC138, CC139) and 9 singleton strains identified in this study are shown in **Figure 3**. All *C. albicans* isolates within CC22, CC125, CC138, and CC139 were from China. CC138 and CC139 are newly reported here. In addition, DST1097, DST2696, DST1865, DST2861, DST448, DST3715, and DST3722 identified in this study were inferred to be the group founders of CC30, CC50, CC18, CC52, CC125, CC138, and CC139, respectively. DST1997 and DST1220 were the sub-group founders of CC9.

## 4 Discussion

Our study comprised 133 patients with candidemia (mean age: 61.75 years; 66.2% male), all of whom presented with multiple comorbidities and risk factors, consistent with previous reports from China (Cheng et al., 2005; Pu et al., 2017; Li et al., 2020a). The observed incidence (0.32 per 1, 000 admissions) was comparable to other Chinese data (0.38 per 1, 000) but significantly lower than rates reported in Asia (Tan et al., 2015), Latin America (Nucci et al., 2013b), and Italy (Tortorano et al., 2013). Although *C. albicans* remained a frequently isolated pathogen, NAC species collectively exhibited higher prevalence, led by *C. parapsilosis*, *C. tropicalis*, and *C. glabrata* in descending order. This pattern contrasts with some Asian reports, where the detection rates of *C. tropicalis*, *C. glabrata*, and *C. parapsilosis* follow a descending order (Tan et al., 2015). The 30-day all-cause mortality was 48.1%, exceeding both global benchmarks (Rodriguez et al., 2017a) and previous domestic reports (Li et al., 2016; Zheng et al., 2021). Notably, infection with *C. albicans* was associated with significantly higher mortality than NAC species, likely attributable to older age, higher comorbidity burden, and increased incidence of septic shock (*P* < 0.01), alongside markedly elevated inflammatory markers. These findings align with established predictors of mortality such as advanced age, septic shock, and mechanical ventilation (De Rosa et al., 2015; Bassetti et al., 2015; Barchiesi et al., 2016; Sbrana et al., 2018; Hirano et al., 2018). The intrinsic virulence of *C. albicans* may further contribute to poor outcomes (Arendrup et al., 2002), warranting heightened clinical attention. In contrast to earlier studies linking leukopenia to *C. albicans* infection (Li et al., 2020a), our cohort demonstrated elevated white blood cell counts, neutrophil counts, and red cell distribution width in *C. albicans* cases compared to NAC infections. These discrepancies may reflect variations in host factors, clinical practices, or antifungal stewardship.

Multivariate analysis identified hypoproteinemia, septic shock, and elevated CRP levels as independent risk factors for mortality in *C. albicans* candidemia. Beyond antifungal therapy, higher platelet count was identified as a protective factor—a previously unreported association suggesting a potential role for platelets in host defense against *C. albicans*. Platelets are known to participate in immune regulation during infections (Fitzgerald et al., 2006; Yeaman, 2010; Cox et al., 2011) and have demonstrated protective effects in viral diseases (Iannacone et al., 2008; Loria et al., 2013). For NAC infections, shock and elevated serum urea were independent risk factors for fatal outcome, consistent with urea being an established predictor of sepsis-related mortality (Harazim et al., 2023). Furthermore, CRP and D-dimer showed prognostic utility in *C. albicans* candidemia, whereas urea and total bilirubin were more predictive in NAC infections, highlighting pathogen-specific differences in biomarker applicability. The identified pathogen-specific biomarker profiles further enable early risk stratification, underscoring the necessity of species-specific clinical management.

In recent years, increasing reports of antifungal resistance and treatment failures have raised serious concerns. Despite guideline recommendations favoring echinocandins, azoles remained the most frequently prescribed antifungals in our center. However, azoles demonstrated suboptimal activity against *C. glabrata* and *C. tropicalis*, with noticeable cross-resistance among voriconazole, fluconazole, itraconazole, and posaconazole. These observations are consistent with studies reporting higher azole resistance in these species compared to other *Candida* spp (Pfaller et al., 2014; You et al., 2017b; Jin et al., 2018). Of particular concern is the emergence of echinocandin-resistant *C. glabrata*, previously reported mainly in the United States (Bassetti et al., 2013a; Faria-Ramos et al., 2014; Boonsilp et al., 2021). Although still rare, the rapid increase in resistance is alarming. In our study, the resistance rate to echinocandins in *C. glabrata* was 16.7%, substantially higher than rates reported in U.S. studies (Kartsonis et al., 2005; Alexander et al., 2013; Vallabhaneni et al., 2015). This phenomenon may be attributed to the updated guidelines recommending echinocandins as first-line therapy for candidemia (Pappas et al., 2016), thereby expanding their clinical use and potentially exerting selective pressure on resistant strains. Additionally, the possibility of transmission of resistant clones within the hospital environment cannot be excluded. Amphotericin B retained consistent *in vitro* activity against all isolates, although its clinical utility remains limited by toxicity.

MLST analysis revealed substantial genetic diversity among *C. albicans* isolates. We identified 7 novel alleles and 23 new diploid sequence types (DSTs), designated DST3707–DST3729, accounting for 62.2% of the isolates—all previously unreported in the PubMLST database. This genetic plasticity may stem from the heterozygosity of the *C. albicans* genome and its high frequency of genetic exchange (Ropars et al., 2018). Host-specific pressures may also drive genetic variation influencing virulence phenotypes (Smith and Hickman, 2020). The MLST analysis indeed suggests possible nosocomial transmission, as 50% of isolates grouped into 6 nosocomial clonal complexes. Our epidemiological investigation into the patients associated with the predominant and persistent CC9 complex revealed that a majority of them were hospitalized in the Surgical Ward, with overlapping admission periods. This spatiotemporal clustering strongly supports the likelihood of within-ward transmission. Studies indicate that the hospital environment and healthcare workers may serve as vectors for nosocomial transmission (Strausbaugh et al., 1994; Reagan et al., 1995; Viviani et al., 2006), highlighting the imperative for enhanced environmental surveillance and strict hand hygiene compliance (Cliff et al., 2008; Tsai et al., 2015). Nearly one-quarter of the DSTs were identified as group or sub-group founders, indicating that genomic adaptability may facilitate the emergence of hospital-adapted lineages. The clonal complexes CC22, CC125, CC138, and CC139 (the latter two being newly identified) demonstrated geographic clustering within China, highlighting the imperative for continued surveillance of their distribution and evolution.Several limitations should be considered. First, the single-center design may limit generalizability. Second, no correlation was established between molecular genotypes and clinical outcomes. Finally, strain attrition reduced the number of isolates available for comprehensive antifungal susceptibility and MLST analysis.

## 5 Conclusions

This study demonstrates that candidemia continues to pose a significant threat with high mortality. The distinct risk factors and prognostic biomarkers identified for *C. albicans* and NAC species underscore the necessity of species-specific clinical management. While antifungal resistance remains uncommon overall, the emergence of azole non-susceptibility in key species highlights the imperative for ongoing susceptibility surveillance. Furthermore, MLST uncovered a highly diverse and evolving population of *C. albicans*, including novel genetic lineages. These findings collectively affirm that integrating robust molecular epidemiology with clinical data is crucial for advancing the global management and surveillance of candidemia.

## Data Availability

The datasets presented in this study can be found in online repositories. The names of the repository/repositories and accession number(s) can be found in the article/supplementary material.
